# Dyrk1a Phosphorylation of *α*-Synuclein Mediating Apoptosis of Dopaminergic Neurons in Parkinson's Disease

**DOI:** 10.1155/2023/8848642

**Published:** 2023-07-10

**Authors:** Yuxuan Yong, Qinfen Wu, Xinling Meng, Ranran Lu, Huan Xia, Feifei Pei, Xinling Yang

**Affiliations:** ^1^The Second Affiliated Hospital of Xinjiang Medical University, Urumqi, Xinjiang 830054, China; ^2^The Fourth Affiliated Hospital of Xinjiang Medical University, Urumqi, Xinjiang 830054, China; ^3^The Third Affiliated Hospital of Xinjiang Medical University, Urumqi, Xinjiang 830054, China

## Abstract

**Objective:**

To investigate the role of aberrant Dyrk1a expression in phosphorylation modification at the *α*-synuclein serine 129 (Ser129) site to analyze its molecular mechanism in mediating apoptosis of PD.

**Methods:**

The protein level of P-*α*-synuclein (Ser129), *α*-synuclein, Bcl-2, Bax, active caspase 3, GSK3*β*, PI3K, AKT, and cyclinD1 were detected. The mRNA transcript levels of Dyrk1a and DAT and protein levels of IL-1*β*, IL-6, COX-2, and TNF-*α* were detected.

**Results:**

P-*α*-synuclein (Ser129), *α*-synuclein, Bax, active caspase 3, GSK3*β*, and cyclinD1 expressions were decreased in Dyrk1a-AAV-ShRNA (*P* < 0.05), and Bcl-2, AKT, and PI3K expressions were increased (*P* < 0.05). Increased TH protein expression was shown in Dyrk1a-AAV-ShRNA (*P* < 0.05). Dyrk1a mRNA was decreased in the Dyrk1a-AAV-ShRNA group (*P* < 0.05), and DAT mRNA was increased (*P* < 0.05). IL-1*β*, IL-6, COX-2, and TNF-*α* protein levels were decreased in Dyrk1al-AAV-Sh-RNA (*P* < 0.05). Transcriptome sequencing showed that Fam220a, which was expected to activate STAT family protein binding activity and participate in the negative regulation of transcription through RNA polymerase II and protein dephosphorylation showed differentially upregulated expression. The untargeted metabolome showed that the major compounds in the Dyrk1a-AAV-ShRNA group were hormones and transmission mediators and the most metabolism-related pathways. Fam220a showed differentially upregulated expression, and differentially expressed genes were enriched for the neuroactive ligand-receptor interaction, vascular smooth muscle contraction, and melanogenesis-related pathways.

**Conclusion:**

Abnormal Dyrk1a expression can affect *α*-synuclein phosphorylation modifications, and dyrk1a knockdown activates the PI3K/AKT pathway and reduces dopaminergic neuron apoptosis. It provides a theoretical basis for the group to further investigate the molecular mechanism.

## 1. Introduction

Parkinson's disease (PD) is an age-dependent neurodegenerative disease with a prevalence of approximately 1.7% in people over 65 years of age [1–3]. The pathogenesis of PD remains unclear, and a multiple-hit hypothesis of PD pathogenesis has been proposed, including genetic and environmental factors, and it simultaneously affects neuronal homeostasis, leading to progressive neurodegeneration of dopaminergic neurons [4]. The degenerative necrosis of dopaminergic neurons in the substantia nigra in PD patients results in the formation of Lewy bodies (LBs) and Lewy neurites (LNs) [5]. LBs and LNs are mainly composed of misfolded a-synuclein, which is one of the key proteins in the pathogenesis of PD. Phosphorylation of the a-synuclein Ser129 site induces an unfolded protein response (UPR) in aa-synuclein, which ultimately leads to functional degenerative necrosis of DA neurons and affects tyrosine hydroxylase (TH) activity [6, 7]. The study of a-synuclein phosphorylation mechanisms has become a research hotspot in recent years. This result suggests that the phosphorylation level of a-synuclein Ser129 site may have an important correlation with the production of LBS and even dopaminergic neuronal degeneration [8, 9]. The phosphorylation process of Ser129 is dynamic, and its phosphorylated form affects the subcellular assignment of PD-associated mutant genes such as A30P and A53T [10–12]. Furthermore, it has been suggested that the binding and dissociation of synaptosomal membranes of mutant a-synuclein may be regulated by pathological Ser129 phosphorylation [13].

The distribution pattern of Dyrk1a in the human brain is specific to the brain region, cell type, and subcellular compartment. Dyrk1a was nearly identical in the frontal, temporal, and occipital cortices. Immunocytochemistry detected significant differences in the brain structure-specific and neuron-type-specific distribution of Dyrk1a, suggesting that the role of Dyrk1a in development, maturation, aging, and degeneration may differ across brain structures and neuronal types [14–16]. Dyrk1a has been shown to have multiple biological functions, as reflected in its interactions with numerous cytoskeletal, synaptic, and nuclear proteins, including transcription factors and splicing factors [17, 18]. Dyrk1a is described as a regulator of a broad spectrum of neurodevelopmental mechanisms that identifies 239 genes deregulated by the overexpression of Dyrk1a through the REST/NRSF chromatin remodeling complex, which suggests a central role for this kinase in brain pathology. The expression of Dyrk1a in fetal and postnatal neurons, as well as in adult and elderly neuron expression, suggests that the regulation of Dyrk1a is an integral part of neuronal development, maturation, and aging [19]. Dyrk1a plays a key role in neuroproliferation and neurogenesis in the developing brain, and its gene is located on chromosome 21 (21q22.2), a region known as the Down syndrome critical region (DSCR) [20, 21]. Due to its location, triplication of the Dyrk1a locus in Down syndrome (DS) results in a 1.5-fold increase in Dyrk1a in the fetal and adult brain [22]. In addition, upregulation of Dyrk1a has been reported to promote altered neuronal proliferation in DS patients through specific phosphorylation of p53 at the Ser15 locus [23].

To date, 1-methyl-4-phenyl-1,2,3,6-tetrahydropyridine (MPTP) and 6-hydroxydopamine (6-OHDA) are by far the most commonly used neurotoxins to cause a Parkinsonian state by causing severe loss of dopaminergic neurons [24, 25]. The gold standard model for motor symptoms of Parkinson's disease was generated by administering MPTP to the Eastern Hemisphere nonhuman primate (NHP) species [26]. The MPTP-treated NHP model is most often replicated after systemic injection of MPTP, which readily crosses the blood-brain barrier and leads to Parkinson's syndrome, manifested by prolonged rest, bradykinesia, postural deficits, and reduced overall movement [27]. MPTP has a broad neurotoxic effect on dopaminergic, adrenergic, and 5-hydroxytryptaminergic neurons outside the substantia nigra. Based on the previous experiments, Dyrk1a gene knockdown phosphorylation modifies a-synuclein and downregulates P-a-synuclein (Ser129) expression. Dyrk1a has direct interaction with P-a-synuclein (Ser129) and GSK-3b, and Dyrk1a gene overexpression inhibits the PI3K/AKT pathway to promote DA neuronal apoptosis. It is suggested that Dyrk1a phosphorylation of a-synuclein mediates apoptosis in dopaminergic neurons. In the study, MPTP-induced PD model mice with tyrosine hydroxylase (TH) as the promoter of adeno-associated virus specifically silenced the expression of Dyrk1a gene in the midbrain substantia nigra by stereotaxic localization in the striatum of the mouse. It was verified by behavioral tests, Western blot (WB) technique, immunohistochemistry, immunofluorescence, enzyme-linked immunosorbent assay (ELISA), HE staining, and transmission electron microscopy to further analyze the molecular mechanism of Dyrk1a phosphorylation of a-synuclein-mediated apoptosis of dopamine neurons.

## 2. Materials and Methods

### 2.1. Experimental Animals

Sixty 6–8-week-old C57BL/6 male mice weighing 25.20 ± 2.12 g were fed on a 12-hour alternating light/dark cycle at 22.10 ± 1.03°C and 60.12 ± 5.09% relative humidity. The experiments were started one week after the mice were acclimated to the animal room environment for modeling. The mice were purchased from the Animal Experiment Center of Xinjiang Medical University, and the experiments were reviewed by the Experimental Animal Ethics Committee of the First Affiliated Hospital of Xinjiang Medical University before the start of the experiments, and the experimental operations and handling processes were in accordance with the ethical standards for experimental animals. Control group (*n* = 15): mice were injected intraperitoneally with 0.9% NaCl, 25 mg/kg, twice a week for five weeks. MPTP group (model, *n* = 15): mice were injected intraperitoneally with MPTP, 25 mg/kg, twice a week for five weeks. Ctrl-AAV-ShRNA (model + MPTP + empty vector, *n* = 15): empty vector (Ctrl-AAV-ShRNA) was injected locally into the striatum of the midbrain of mice, (coordinates: Bregma AP, −3.0 mm, ML, ±1.3 mm, DV, −4.7 mm; dose: 2.85 × 1012 PFU/mL, 0.5 *μ*L per side). Three weeks after stereotactic injection, mice were injected intraperitoneally with MPTP, 25 mg/kg, twice a week for five weeks. Dyrk1a-AAV-ShRNA group (model + MPTP + Dyrk1a-AAV-ShRNA, *n* = 15): Dyrk1a-AAV-ShRNA was first injected stereotactically into the nigrostriatal region of the midbrain of mice, (coordinates: Bregma AP, −3.0 mm, ML, ±1.3 mm, DV, −4.7 mm. 3.43 × 1012 PFU/mL, 0.5 *μ*L per side). Three weeks after stereotactic injection, mice were injected intraperitoneally with MPTP, 25 mg/kg, twice a week for five weeks.

### 2.2. Behavioral Tests

The pole climbing experiment, suspension test, open field experiment, and grasping force experiment were performed.

### 2.3. Western Blot Experiment and qRT-PCR

The protein of each treatment group was extracted. We boiled the upper sample solution in boiling water at 100°C for 5 min to denature the protein and added primary antibody diluted in the blocking solution to the incubation bag and incubated overnight at 4 °C. We washed the membrane 5 min × 3 times with TBST and incubated horseradish peroxidase-labeled sheep anti-rabbit secondary antibody (Jackson 1 : 5000) or horseradish peroxidase-labeled sheep anti-mouse secondary antibody (Jackson 1 : 5000) at room temperature for 2 h. We washed the membrane 15 min × 5 times with TBST. The membrane was made to react with the chemiluminescence detection reagent (Reagent A: Reagent *B* = 1 : 1) for 2 min, the membrane was removed, the excess liquid was shaken off, the PVDF membrane was wrapped with cling film, and the membrane was developed and fixed in the dark room.

Tissue samples were ground with liquid nitrogen, and total RNA was extracted with Trizol for every 50–100 mg of the tissue to use for qRT-PCR. Total RNA was extracted using Trizol (Invitrogen, USA) and changed into cDNA by the using reverse transcriptase kit (Fermentas, USA). The cycling conditions were 95°C for 5 min, followed by 35 cycles of 95°C for 10 s, 50–60°C for 35 s, and 72°C for 30 s. Temperature increases were 72°C for 5 min. The RT-qPCR analysis was performed with the Light Cycler 480 RT-qPCR System (Roche, Basel, Switzerland). Fold changes in gene expression were estimated using the CT comparative method, normalizing GAPDH. CT values relative to control samples are as follows: ΔCt = Ct (assayed samples)-Ct(*β*-actin); ΔΔCt = ΔCt − ΔCt control; and fold difference = 2^−(ΔΔCT)^.

### 2.4. HE Staining

The tissue blocks were taken, fixed, conventionally paraffin-embedded, cut into 4 *μ*m sections, dewaxed in xylene, washed by all levels of ethanol to water, stained with hematoxylin for 5 min, and rinsed with tap water. Hydrochloric acid ethanol fractionation was performed for 30 s along with tap water immersion for 15 min or warm water (about 50°C) for 5 min. The tissue sections were subjected to a series of steps for preparation. First, the sections were treated with eosin solution and left for 2 minutes. Then, conventional dehydration was performed, followed by transparency treatment. The sections were sealed afterwards. Finally, slices were obtained using an orthomosaic microscope, with field of view magnifications of ×10, ×100, ×200, and ×400, respectively.

### 2.5. Immunohistochemical Staining

Immunohistochemical assay (IHC)-baked slices: put in a 68°C incubator about 20 min, tissue rehydration, 3% H2O2 room temperature for 10 min, washed with PBS 3 times, 5 min each time to remove endogenous enzymes, trypsin and dilution solution diluted at 1 : 3 and added dropwise to the tissue, and incubated at 37°C for 10 min. We diluted the primary antibody with the prepared closure solution. The primary antibody (1 : 200 dilution) was incubated overnight at 4°C in the refrigerator. We added the HRP-labeled secondary antibody and incubated for 30 min at 37°C and removed the tissue sections from the 37°C incubator, washed 3 times with PBS for 5 min each time, and absorbed the liquid around the tissue with an absorbent paper. 50 *μ*L of concentrated DBH was mixed with 1 mL of DAB substrate, added dropwise on the tissue, observed the brown shade under the microscope, and rinsed with tap water when the color reaches the optimum. The tissue slices underwent hematoxylin staining, followed by dehydration using a gradient of alcohol. Subsequently, the slices were sealed.

### 2.6. ELISA

The plates were sealed tightly with sealing film and incubated at 37°C for 30 minutes. We diluted the 30-fold concentrated washing solution 30-fold with distilled water and set aside and discarded the sealing membrane, shook off the liquid, filled with washing solution, and repeated this 5 times and pat dry. The enzyme reagent was added 50 *μ*l/per well, except for blank wells. We warmed up, washed, and added 50 *μ*l of the chromogenic agent A and then 50 *μ*l of chromogenic agent B to each well, shook and mixed gently, and developed for 15 minutes at 37°C and avoided light. Then, we added 50 *μ*l of termination solution per well to terminate the reaction.

### 2.7. Transmission Electron Microscopy

The material was taken and fixed with 1% osmium acid prepared in 0.1 M phosphate buffer PB (PH7.4) for 2 h at room temperature avoiding light, rinsed 3 times in 0.1 M phosphate buffer PB (PH7.4), dehydrated at room temperature, osmotically embedded, ultrathin sections, copper mesh stained in 2% uranyl acetate saturated alcohol solution avoiding light for 8 min; washed 3 times in 70% alcohol; washed 3 times in ultrapure water; 2.6% lead citrate solution avoiding carbon dioxide staining for 8 min; washed with ultra-pure water 3 times; and used a filter paper to slightly blot-dry. We observed the material under a transmission electron microscope and collected images for analysis.

### 2.8. Metabolomics Analysis

The sample was precisely measured and extracted, and then the supernatant metabolites were extracted by centrifugation for liquid-liquid mass spectrometry (LC-MS). The chromatographic conditions were as follows: the column was an ACQUITY UPLC HSS T3 (100 mm × 2.1 mm i.d., 1.8 *μ*m; Waters, Milford, USA), mobile phase A was 95% water + 5% acetonitrile (containing 0.1% formic acid), mobile phase B was 47.5% acetonitrile + 47.5% isopropanol + 5% water (containing 0.1% formic acid), and the injection volume was 2 *μ*L. The column temperature was 40°C. The metabolomics software Progenesis QI (WatersCorporation, Milford, USA) was used for peak extraction, alignment, and identification, and the final data matrix contained the retention time, peak area, and mass-to-charge ratio. The information was obtained for postprocessing and raw letter analysis. Differential metabolites were performed. Based on hypergeometric analysis, KEGG enrichment analysis was performed for differential metabolites.

### 2.9. Transcriptome Sequencing

Total RNA was extracted, and the concentration, purity, and RIN were examined using Nanodrop2000 and Agilent2100. The Illumina TruseqTM RNA sample prep kit was used for library construction. Using magnetic beads with oligo (dT), mRNA can be isolated from total RNA. The mRNA can be broken randomly, and small fragments of about 300 bp can be separated by magnetic beads. Six-base random primers are added to synthesize one-stranded cDNA, followed by two-stranded synthesis to form a stable double-stranded structure. The end repair mix is added to make it flat-terminated, followed by the addition of an “A” base at the 3′ end to join the Y-junction. Sequencing was performed based on Illumina Novaseq 6000. Differential gene analysis was performed using the differential analysis software DESeq2, and the differential gene screening criteria were |log2FC| ≥ 1 and *p* value <0.05. Based on hypergeometric analysis, functional enrichment analysis of GO and KEGG was performed for differential genes and *p* < 0.05 was considered as the significant term.

### 2.10. Statistical Analysis

SPSS 22.0 was used for data processing, and GraphPad Prism 9.00 software was used for analysis and graphing. The experimental data were expressed as the mean ± standard deviation, and the data differences between the two groups were analyzed by Student's *t*-test, the variance discrepancy was tested by the tʹ test, the comparison between multiple groups was analyzed by one-way analysis of variance (ANOVA), and the LSD was used to analyze between the two groups. ^*∗*^: *p* < 0.05, ^*∗∗*^: *p* < 0.01, ^*∗∗∗*^: *p* < 0.001 vs. the control group; #: *p* < 0.05, ##: *p* < 0.01, ###: *p* < 0.001 vs. the MPTP group; the differences were statistically significant.

## 3. Results

### 3.1. Phenotype Analysis

**Figure 1:**
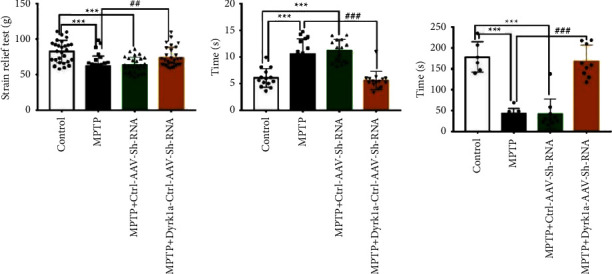
Animal behavioral test ^*∗*^: *P* < 0.05, ^*∗∗*^: *P* < 0.01, and ^*∗∗∗*^: *P* < 0.001 vs. the control group. ^#^: *P* < 0.05, ^##^: *P* < 0.01, ^###^: *P* < 0.001 vs. the MPTP group.

The average pulling force value was 82.69 ± 15.84 g for the control, 62.07 ± 13.94 g for the model, 63.33 ± 11.03 g for Ctrl-AAV-ShRNA, and 74.09 ± 13.87 g for Dyrk1a-AAV-ShRNA. The model group and the Ctrl-AAV-ShRNA group had lower pulling force (*P* < 0.001) (Figure 1(a)), suggesting that Dyrk1a knockdown could increase the grasping force of the limb of mice. The time spent in the control group was 6.1 ± 1.732, the model was 10.57 ± 2.839, Ctrl-AAV-ShRNA was 11.18 ± 2.117, and Dyrk1a-AAV-ShRNA was 5.587 ± 1.71. The difference was statistically significant (*P* < 0.001) in the MPTP + Dyrk1al-AAV-ShRNA group (Figure 1(b)). The suspension time was 178.7 ± 36.5 in the control, 42.63 ± 12.51 in the model, 41.7 ± 35.87 in Ctrl-AAV-ShRNA, and 167.9 ± 38.59 in Dyrk1a-AAV-ShRNA. The differences in MPTP + Dyrk1al-AAV-ShRNA were statistically significant (*P* < 0.001) (Figure 1(c)).

**Figure 2:**
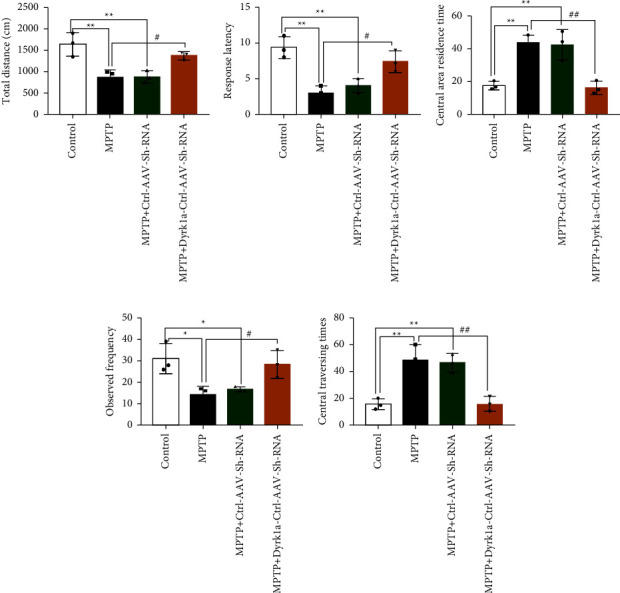
Open field experimental test results: (a) total distance of movement; (b) first frame latency; (c) central area dwell time; (d) number of standing observations; and (e) number of central crossings. Experimental results are expressed as the mean ± standard error ($\bar{X}$*X* ± SD). ^*∗*^: *P* < 0.05, ^*∗∗*^: *P* < 0.01, ^*∗∗∗*^: *P* < 0.001 vs. the control group. ^#^: *P* < 0.05, ^##^: *P* < 0.01, and ^###^: *P* < 0.001 vs. the MPTP group.

Compared with the model group, the total distance of movement was significantly increased in MPTP + Dyrk1al-AAV-Sh-RNA (*P* < 0.05) (Figure 2(a)). The latency within the first frame was significantly higher in the MPTP + Dyrk1al-AAV-Sh-RNA group (*P* < 0.05) (Figure 2(b)). The dwell time in the central region of the MPTP + Dyrk1al-AAV-Sh-RNA group was significantly lower (*P* < 0.01) (Figure 2(c)). The results of the number of standing observations showed that the number of standing observations was significantly increased in MPTP + Dyrk1al-AAV-Sh-RNA (*P* < 0.05) (Figure 2(d)). We observed the activity of mice in the central area in each group and found that the model and Ctrl-AAV-ShRNA were less likely to stay or traverse in the central area and MPTP + Dyrk1al-AAV-ShRNA was more active and traversed the central area (*P* < 0.01) (Figure 2(e)).

### 3.2. Pathological Analysis

**Figure 3:**
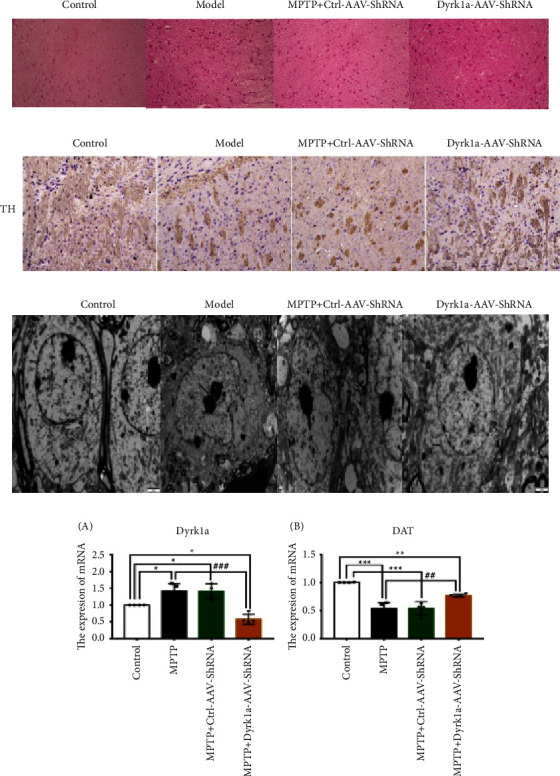
(a): HE staining of the brain tissue of mice in each group (×200); (b) immunohistochemical staining; (c) ultrastructural alterations in the nigrostriatal cells of the mouse midbrain; and (d) detection of Dyrk1a and DAT expression by qRT-PCR, ^*∗*^: *P* < 0.05, ^*∗∗*^: *P* < 0.01, and ^*∗∗∗*^: *P* < 0.001 vs. the control group. ^#^: *P* < 0.05, ^##^: *P* < 0.01, and ^###^: *P* < 0.001 vs. the MPTP group.

The brain tissue of Ctrl-AAV-Sh-RNA showed that vacuolation, neuronal atrophy, individual inflammatory cells, and neuronal degeneration were improved in MPTP + Dyrk1a-AAV-ShRNA and no inflammatory cells were present (Figure 3(a)). TH was positively expressed in MPTP + Dyrk1a-AAV-ShRNA (Figure 3(b)). In the Ctrl-AAV-ShRNA group, neuronal cytosolic organelles were reduced, mitochondria were swollen and deformed, vacuoles appeared in the cytoplasm, the nucleus was obviously wrinkled, and the nucleolus density was increased (Figure 3(c)). In the MPTP + Dyrk1al-AAV-Sh-RNA group, nigrostriatal neurons were cytosolic. The damaged neurons entered the recovery period, the nucleus morphology of the neurons was close to normal, the mitochondrial morphology was improved than before, and the number of increased coarse endoplasmic reticulum was gradually restored (Figure 3(c)).

Dyrk1a was elevated in the model and Ctrl-AAV-ShRNA and decreased in MPTP + Dyrk1a-AAV-ShRNA (*P* < 0.05). DAT was increased in MPTP + Dyrk1a-AAV-ShRNA (*P* < 0.01) (Figure 3(d)).

### 3.3. AKT, PI3K, and Inflammatory Marker Analysis

**Figure 4:**
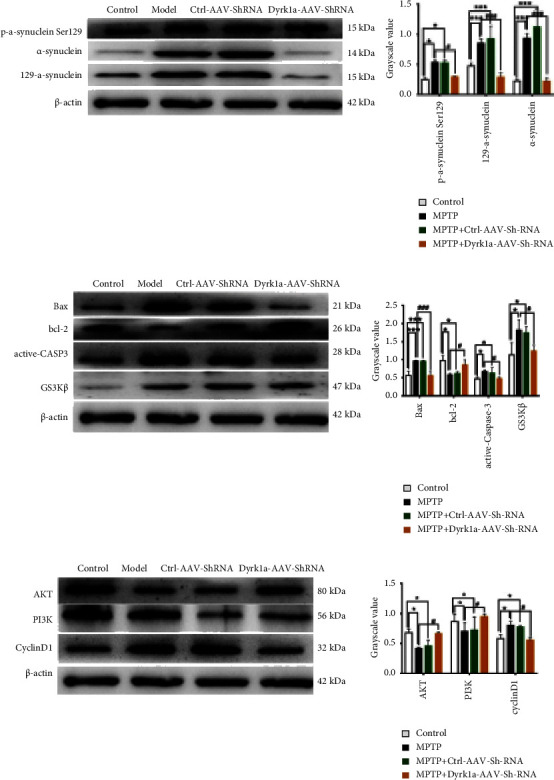
A WB expression levels. ^*∗*^*P* < 0.05, ^*∗∗*^: *P* < 0.01, and ^*∗∗∗*^: *P* < 0.001 vs. the control group. ^#^: *P* < 0.05, ^##^: *P* < 0.01, and ^###^: *P* < 0.001 vs. the MPTP group.

Compared with the model group, p-a-synuclein Ser129, 129-a-synuclein, a-synuclein, Bax, active caspase 3, and cyclinD1 expressions were decreased in the MPTP + Dyrk1a-AAV-ShRNA group (*P* < 0.05), and the expressions of Bcl-2, AKT, and PI3K were increased (*P* < 0.05, Figures 4(a)–4(c)).

**Figure 5:**
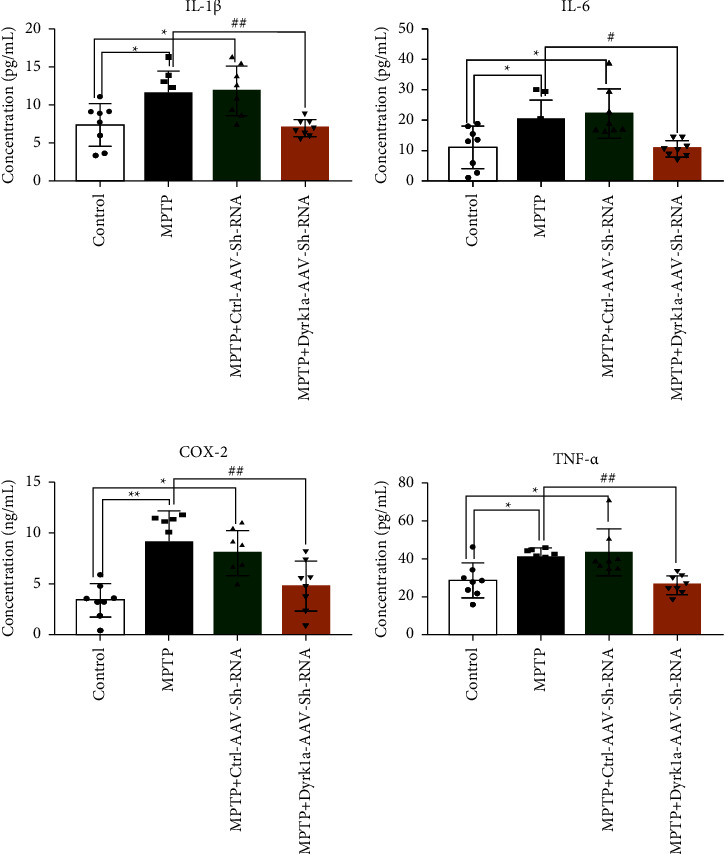
Expression levels of IL-1*β*, IL-6, COX-2, and TNF-*α* by ELISA. ^*∗*^: *P* < 0.05, ^*∗∗*^: *P* < 0.01, and ^*∗∗∗*^: *P* < 0.001 vs. the control group. ^#^: *P* < 0.05, ^##^: *P* < 0.01, and ^###^: *P* < 0.001 vs. the MPTP group.

The expression levels of IL-1*β*, IL-6, COX-2, and TNF-*α* were decreased in the MPTP + Dyrk1al-AAV-Sh-RNA group compared with the model group (*P* < 0.05, *P* < 0.01) (Figures 5(a)–5(d)).

### 3.4. Differential Metabolite Analysis

**Figure 6:**
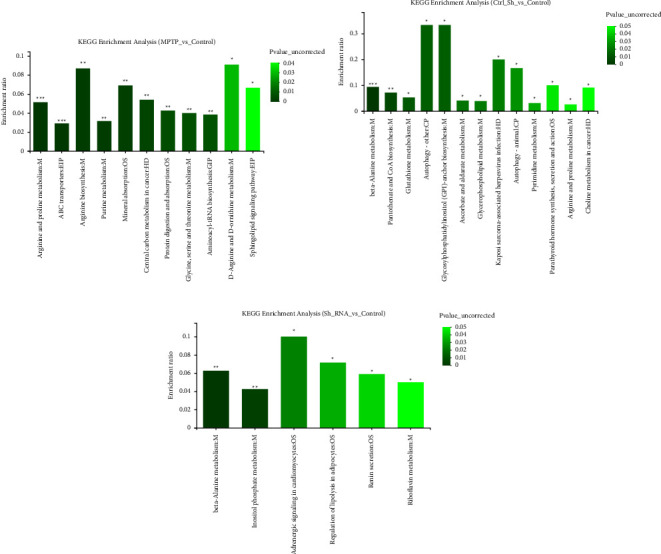
Enrichment analysis of KEGG for differential metabolites.

A total of 36 metabolites were differentially expressed in the model group compared to the control group; 63 metabolites were differentially expressed in Ctrl-AAV-ShRNA; 46 metabolites were differentially expressed in Dyrk1a-AAV-ShRNA (Figure 6(a)). Propionylcarnitine, *N*-acetyl-L-glutamic acid, and 6-((2-carboxyacetyl)oxy)-3,4,5-trihydroxyoxane-2-carboxylic acid were significantly upregulated in Ctrl-AAV-ShRNA (*P* < 0.5) and tolmetin glucuronide, lysoPC (18 : 1(9Z), and nicotinate beta-D-ribonucleotide were significantly downregulated (*P* < 0.5). Differential metabolite was significantly enriched in autophagy-other, glycosylphosphatidylinositol (GPI)-anchor biosynthesism, Kaposi sarcoma-associated herpesvirus infection, and autophagy animal pathways (Figure 6(b)).

Dihydrocapsaicin, riboflavin, vitamin B2, and D-erythrose 4-phosphate were significantly upregulated in Dyrk1a-AAV-ShRNA (*P* < 0.5), and adenine, berteroin, and D-myo-inositol 1,4-bisphosphate were significantly downregulated (*P* < 0.5). Differential metabolite was significantly enriched in adrenergic signaling in cardiomyocytes, beta-alanine metabolism, and regulation of lipolysis in adipocytes; the pathways were related to the regulation of lipolysis in adipocytes and renin secretion (Figure 6(c)).

### 3.5. Differential Gene Analysis

There were 215 differentially expressed genes in Ctrl-AAV-ShRNA, including 142 upregulated and 73 downregulated genes. Significantly upregulated expressed genes in Ctrl-AAV-ShRNA were Ighv1-19, Gm20482, and Ighg2b and downregulated genes were Gstp2, Emx1, and Cltrn (Table 1).

**Figure 7:**
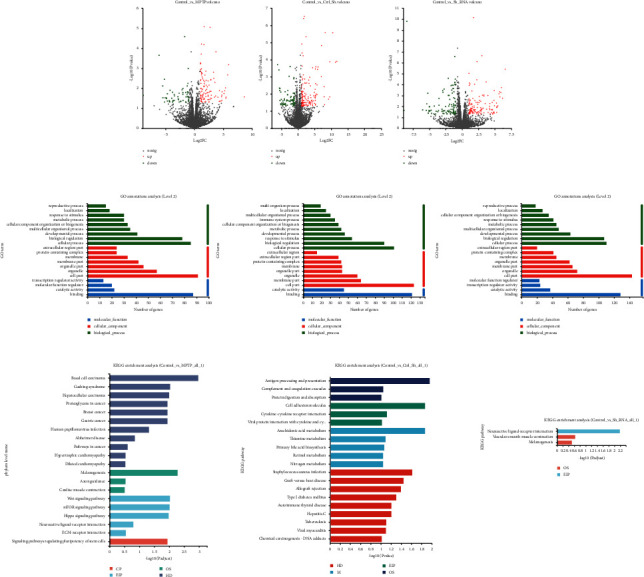
The volcano and enrichment analysis of GO and KEGG for differential genes.

Functional annotation analysis of GO and KEGG was performed for the differential genes (Figure 7(a)). Differentially expressed genes in Ctrl-AAV-ShRNA has mainly cellular process, biological regulation, response to stimulus, developmental process, and metabolic process in the biological process (Figure 7(b)). Differential genes of Ctrl-AAV-ShRNA were mainly enriched in antigen processing and presentation, complement and coagulation cascades, and protein digestion and absorption pathways in the organism system. In environmental information processing were cell adhesion molecules, cytokine-cytokine receptor interaction, and viral protein interaction with cytokine and cytokine receptors. Metabolism-related pathways were enriched for arachidonic acid metabolism, thiamine metabolism, primary bile acid biosynthesis, retinol metabolism, and nitrogen metabolism pathways. (Figure 7(c)).

The MPTP + Dyrk1a-AAV-ShRNA group had 228 differentially expressed genes, including 131 upregulated and 97 downregulated genes. The differentially upregulated expression genes in Dyrk1a-AAV-ShRNA were Fam220a, which was expected to activate STAT family protein binding activity and to be involved in negative transcriptional regulation through RNA polymerase II and protein dephosphorylation. Differentially downregulated expression gene was Tmem215, which maintained endothelial cell survival to promote angiogenesis. Differentially expressed genes in Dyrk1a-AAV-ShRNA had mainly cellular process, biological regulation, developmental process, and multicellular organismal process. The differential genes in Dyrk1a-AAV-ShRNA were enriched for the neuroactive ligand-receptor interaction, vascular smooth muscle contraction, and melanogenesis-related pathways (Figure 7(c)).

## 4. Discussion

PD is a progressive neurodegenerative disorder that affects approximately 1% of the population over 55 years of age, with the highest prevalence in people aged 85 years and older [28]. The clinical manifestations of PD are due to extensive loss of dopaminergic neurons in the nigrostriatal pathway and neuronal dysfunction in the dopaminergic, 5-hydroxytryptaminergic, adrenergic, and cholinergic neurotransmitter systems [29]. Both metabolites and proteins reflect the physiological and pathological state of the individual. Analysis of these data types may help identify sensitive and effective markers for early disease detection [30]. Andrea et al. performed ultrahigh performance liquid chromatography mass spectrometry analysis of plasma from 21 patients with PD and suggested that tyramine could be used as a marker for early PD and suggested that tyramine, noradrenaline, and tyrosine could be used as prognostic markers [31, 32]. Wichter et al. performed a high-performance liquid chromatography study of monoamines in the plasma of patients with PD and found a significantly higher homovanillic acid/dopamine ratio and a lower 5-hydroxyindoleacetic acid/5-hydroxytryptamine ratio, emphasizing the involvement of multiple neurotransmitter systems in the disease [33, 34]. Sonnien et al. observed increased production of LRRK2 mutant synaptophysin by astrocytes from *α*-mutant PD patients, altered metabolism and calcium homeostasis, increased cytokine release, increased levels of polyamines and their precursors, and decreased levels of lysophosphatidylethanolamine [35]. These findings suggest that astrocytes may be involved in the pathogenesis of PD.

Understanding the altered metabolic pathways and metabolites involved in the development and progression of the disease can help better understand the underlying relevant biological alterations. In this study, brain tissue samples from a mouse model of MPTP-induced chronic Parkinson's disease were metabolomically characterized by an ultra-performance liquid chromatography mass spectrometry untargeted metabolome. Compared with the control group, the differential metabolic pathways in Ctrl-AAV-ShRNA were metabolism-related pathways, in which the metabolic pathways of cofactors and vitamins, amino acid metabolism, other amino acid metabolism, and carbohydrate metabolism-related pathways were dominant. The major compounds in the Dyrk1a-AAV-ShRNA group were hormones and transmission mediators. The Dyrk1a-AAV-Sh-RNA group had the most metabolism-related pathways, among which the metabolic pathways of cofactors and vitamins and carbohydrate metabolism-related pathways were dominant.

In this experiment, brain tissue samples were sequenced by transcriptome sequencing. The differentially upregulated expression genes in the Dyrk1a-AAV-ShRNA group were Fam220a, which were expected to activate STAT family protein binding activity and were expected to be involved in negative transcriptional regulation through RNA polymerase II and protein dephosphorylation. Differential downregulated expression of gene Tmem215, which maintains endothelial cell survival to promote angiogenesis. The results of KEGG pathway analysis showed that the differential genes in the model were mainly enriched in human disease-related pathways, which were basal cell carcinoma, Cushing's syndrome, and epithelial cell carcinoma. Enriched in environmental information processing-related pathways are the Wnt signaling pathway and the mTOR signaling pathway. Differential genes in Dyrk1a-AAV-ShRNA were enriched for pathways related to neuroactive ligand-receptor interactions, vascular smooth muscle contraction, and melanogenesis.

## 5. Conclusion

In PD model mice with low specific knockdown of DAergic neurons in the midbrain Dyrk1a gene, P- *α*-synuclein (Ser129), *α*-synuclein, Bax, active caspase 3, GSK3*β*, and cyclinD1 expressions were decreased in Dyrk1a-AAV-ShRNA and Bcl-2, AKT, and PI3K expressions were increased. IL-1*β*, IL-6, COX-2, and TNF-*α* protein levels were decreased. The untargeted metabolome showed that the major compounds in the Dyrk1a-AAV-ShRNA group were hormones and transmission mediators and the most metabolism-related pathways. Transcriptome sequencing showed that Fam220a, which was expected to activate STAT family protein binding activity and participate in negative regulation of transcription, showed differentially upregulated expression. Differentially expressed genes were enriched for the neuroactive ligand-receptor interaction, vascular smooth muscle contraction, and melanogenesis-related pathways. Abnormal Dyrk1a expression can affect *α*-synuclein phosphorylation modifications, and dyrk1a knockdown activates the PI3K/AKT pathway and reduces dopaminergic neuron apoptosis. This study required further functional validation experiments and verification of its clinical significance, and further analysis was also needed.

## Figures and Tables

**Table 1:** The differential gene.

Gene id	Gene name	Gene description	Control vs MPTP
ENSMUSG00000033726	Emx1	Empty spiracles homeobox 1	yes|up
ENSMUSG00000059493	Nhs	NHS actin remodeling regulator	yes|down
ENSMUSG00000058620	Adra2b	Adrenergic receptor, alpha 2b	yes|up
ENSMUSG00000015401	Cltrn	Collectrin, amino acid transport regulator	yes|down
ENSMUSG00000083012	Fam220a	Family with sequence similarity 220, member A	yes|up
ENSMUSG00000045005	Fzd5	Frizzled class receptor 5	yes|up
ENSMUSG00000075334	Rprm	reprimo,TP53dependent G2 arrest mediator candidate	yes|up
ENSMUSG00000040253	Gbp7	Guanylate binding protein 7	yes|down
ENSMUSG00000109228	Fam81b	Family with sequence similarity 81, member B	yes|down
ENSMUSG00000110696	Gm45706	Predicted gene 45706	yes|down
Gene id	Gene name	Gene description	Control vs ctrl sh
ENSMUSG00000096410	Ighv1-19	Immunoglobulin heavy variable V1-19	yes|up
ENSMUSG00000092490	Gm20482	Predicted gene 20482	yes|up
ENSMUSG00000038155	Gstp2	Glutathione S-transferase, pi 2	yes|down
ENSMUSG00000033726	Emx1	Empty spiracles homeobox 1	yes|down
ENSMUSG00000015401	Cltrn	Collectrin,amino acid transport regulator	yes|down
ENSMUSG00000076613	Ighg2b	Immunoglobulin heavy constant gamma 2B	yes|up
ENSMUSG00000094335	Igkv1-117	Immunoglobulin kappa variable 1–117	yes|up
ENSMUSG00000003379	Cd79a	CD79A antigen (immunoglobulin-associated alpha)	yes|up
ENSMUSG00000030680	Pagr1a	PAXIP1 associated glutamate rich protein 1A	yes|down
ENSMUSG00000079466	Prdm12	PR domain containing 12	yes|down
Gene id	Gene name	Gene description	Control vs sh RNA
ENSMUSG00000046593	Tmem215	Transmembrane protein 215	yes|down
ENSMUSG00000015401	Cltrn	Collectrin, amino acid transport regulator	yes|down
ENSMUSG00000083012	Fam220a	Family with sequence similarity 220, member A	yes|up
ENSMUSG00000046668	Cxxc5	CXXC finger 5	yes|down
ENSMUSG00000097029	Gm26560	Predicted gene,26560	yes|down
ENSMUSG00000030680	Pagr1a	PAXIP1-associated glutamate rich protein 1A	yes|down
ENSMUSG00000045005	Fzd5	Frizzled class receptor 5	yes|up
ENSMUSG00000050700	Emilin3	Elastin microfibril interfacer 3	yes|up
ENSMUSG00000097093	C330013E15Rik	RIKEN cDNA C330013E15gene	yes|up
ENSMUSG00000038765	Lmx1b	LIM homeobox transcription factor 1 beta	yes|up

Note: gene id, gene name; gene description, the differential genes in the comparison group; yes indicates significant difference, up is upregulation, and down is down-regulation.

## Data Availability

The data used to support the findings of this study are included within the article.
